# Hypertonic saline attenuates expression of Notch signaling and proinflammatory mediators in activated microglia in experimentally induced cerebral ischemia and hypoxic BV-2 microglia

**DOI:** 10.1186/s12868-017-0351-6

**Published:** 2017-03-14

**Authors:** Wen-Xin Zeng, Yong-Li Han, Gao-Feng Zhu, Lin-Qiang Huang, Yi-Yu Deng, Qiao-Sheng Wang, Wen-Qiang Jiang, Miao-Yun Wen, Qian-Peng Han, Di Xie, Hong-Ke Zeng

**Affiliations:** Department of Emergency and Critical Care Medicine, Guangdong General Hospital, Guangdong Academy of Medical Sciences, Guangzhou, 510080 People’s Republic of China

**Keywords:** Cerebral ischemia, Hypertonic saline, Microglia, Notch signaling, Neuroinflammation

## Abstract

**Background:**

Ischemic stroke is a major disease that threatens human health in ageing population. Increasing evidence has shown that neuroinflammatory mediators play crucial roles in the pathophysiology of cerebral ischemia injury. Notch signaling is recognized as the cell fate signaling but recent evidence indicates that it may be involved in the inflammatory response in activated microglia in cerebral ischemia. Previous report in our group demonstrated hypertonic saline (HS) could reduce the release of interleukin-1 beta and tumor necrosis factor-alpha in activated microglia, but the underlying molecular and cellular mechanisms have remained uncertain. This study was aimed to explore whether HS would partake in regulating production of proinflammatory mediators through Notch signaling.

**Results:**

HS markedly attenuated the expression of Notch-1, NICD, RBP-JK and Hes-1 in activated microglia both in vivo and in vitro. Remarkably, HS also reduced the expression of iNOS in vivo, while the in vitro levels of inflammatory mediators Phos-NF-κB, iNOS and ROS were reduced by HS as well.

**Conclusion:**

Our results suggest that HS may suppress of inflammatory mediators following ischemia/hypoxic through the Notch signaling which operates synergistically with NF-κB pathway in activated microglia. Our study has provided the morphological and biochemical evidence that HS can attenuate inflammation reaction and can be neuroprotective in cerebral ischemia, thus supporting the use of hypertonic saline by clinicians in patients with an ischemia stroke.

**Electronic supplementary material:**

The online version of this article (doi:10.1186/s12868-017-0351-6) contains supplementary material, which is available to authorized users.

## Background

Stroke, including ischemic stroke and hemorrhagic stroke, is a major cause of long-term disability and death throughout the world and leads to heavy socioeconomic pressures. An epidemiology study has reported that up to 67.3–80.5% of stroke cases are attributed to ischemic stroke [1]. Furthermore, about 2–8% of all hospitalized patients with ischemic stroke are caused by middle cerebral artery (MCA) infarction, and the risk of neurological deterioration and death is as high as 40–80% [2]. Increasing evidence has shown that neuroinflammatory mediators, which exert deleterious effects and exacerbate the progression of tissue damage, play crucial roles in the pathophysiology of cerebral ischemia injury [3, 4]. A variety of inflammatory mediators, such as interleukin-1β (IL-1β), tumor necrosis factor-α (TNF-α), inducible nitric oxide synthase (iNOS), nitric oxide (NO), reactive oxygen species (ROS) are widely thought to be derived from activated microglia, which is the main resident immune cells within the central nervous system (CNS) that play an important role in the development of inflammatory response after cerebral ischemic insults [5–7]. Therefore, it seems to be a progressing therapeutic strategy to suppress the excessive inflammatory mediators driven by activated-microglia in cerebral ischemia.

There is ample evidence showing that Notch signaling plays an important role in regulating the activation of immune cells such as T cells and macrophages [8–10]. In CNS, Notch signaling is recognized as the cell fate signaling which is activated when Notch receptor binds to its ligand, thus promoting two proteolytic cleavage events at the receptor. The second cleavage by γ-secretase enzyme complex releases the Notch intracellular domain (NICD), which then translocates into the nucleus where it binds to recombination signal sequence-binding protein J (RBP-JK), causing the transcriptional activation of Notch targeting genes including the Hairy-Enhancer of Split (HES) and HES-related proteins (HERP) genes [11–13]. However, recent evidence indicates that Notch signaling may participate in inflammatory response in activated microglia in cerebral ischemia; Indeed, inhibition of Notch signaling resulted in reduced cell numbers of activated microglia, decreased expression of proinflammatory cytokines and cerebral infarct size and improved functional outcome in a model of focal ischemic stroke [14, 15]. Moreover, in vivo and in vitro studies have shown that Notch signaling can regulate NF-κB pathway which has been widely thought to play a key role in inflammtory response [16, 17]. In light of the above, Notch signaling may be a novel and important target to reduce inflammatory response and thus improve the function in ischemic stroke.

In clinic, hypertonic saline (HS), which has been shown to effectively reduce intracranial pressure and hemispheric edema, is widely utilized in acute brain injuries including ischemic stroke, traumatic brain injury and intracranial hemorrhage [2, 18, 19]. To date, many reports have indicated that HS has neuroprotective properties and could reduce mortality after cerebral ischemia [20–22]. Even though the osmotic property of HS has been well studied in brain injury, its extraosmotic effect is still unclear and is being widely investigated. HS was reported to prevent the activation of macrophages and NF-κB pathway and reduce the inflammatory response [23–25]. Previous study in our group demonstrated that HS could reduce infarct size and decrease the release of neuroinflammatory mediators, IL-1β and TNF-α in activated microglia in vivo and in vitro [26]. However, the underlying molecular and cellular mechanisms of HS on inflammation response in activated microglia within cerebral ischemia have remained uncertain. This study was aimed to determine whether HS would partake in regulating production of proinflammatory mediators through Notch signaling in activated microglia in experimentally induced middle cerebral artery occlusion (MCAO) model of ischemic stroke and BV-2 microglial cells activated by oxygen glucose deprivation.

## Methods

### Animals and experimental groups

Adult male Sprague-Dawley (SD) rats weighing 220–250 g were used for *in vivo* experiments. Transient focal ischemia was induced by MCAO model (2 h) using an intraluminal suture method. Rats were randomly divided into sham operated group (Sham group), MCAO group, MCAO + normal saline treatment group (NS group) and MCAO + 10% HS treatment group (10% HS group). After anesthetized achieved with an intraperitoneal injection of 10% chloral hydrate, rats in the sham group were subjected to the surgical procedures at the right common carotid artery (CCA) without occlusion, while those in the other three groups were subjected to right-sided middle cerebral artery occlusion (MCAO) using an intraluminal suture approach. Two hours after MCAO treatment, the rats in NS and HS group were continuously injected, respectively, with normal saline (0.3 ml/h) or 10% HS (0.3 ml/h) by intravenous infusion via the tail vein until the end of the experiment. The rats in sham group, MCAO group, NS group and 10% HS treatment group were further subdivided into two subgroups according to different treatment time points: 12 and 24 h.

### Model of cerebral ischemia

Rats were fasted with only access to water for overnight before subjected to the surgical procedure. Cerebral ischemia was induced by middle cerebral artery occlusion as described previously [27], but with some modifications. In brief, the rats were anesthetized with 10% chloral hydrate followed by a ventral midline incision at the neck. CCA, external carotid artery (ECA) and internal carotid artery (ICA) were exposed and carefully blunt dissected free from the adjacent vagus nerve. Next, the ICA was temporally clipped, the ECA and the CCA were ligated and an arteriotomy was made proximally to the carotid bifurcation. A head-end spherical nylon suture was inserted through the arteriotomy and advanced into the ICA 17–19 mm beyond the carotid bifurcation. The suture, which reached the origin of the middle cerebral artery (MCA), was ascertained by a mild resistance felt. At 2 h post-MCAO, the intraluminal suture was withdrawn to allow reperfusion. Following this, the neurologic deficits were scored on a five-point scale: 0, no neurologic deficit; 1, a mild focal neurologic deficit (failure to extend left forepaw fully); 2, a moderate focal neurologic deficit (circling to the left); and 3, a severe focal deficit (falling to the left); 4, no spontaneous motor activity (the rats did not walk spontaneously and had a depressed level of consciousness). The rats with neurologic deficit score of 0 and 4 were excluded from the study for further analysis.

### Cell culture and treatment

BV-2 microglial cells received from Southern Medical University and identified by lectin (Sigma, St. Louis, MO, USA; Cat. No. L0401) were cultured in Dulbecco’s modified Eagle’s medium-F12 nutrient mixture (DMEM-F12) (Invitrogen Life Technologies Corporation, Carlsbad, CA, USA; Cat. No. 31330-038) supplemented with 10% fetal bovine serum (FBS) (Invitrogen Life Technologies Corporation; Carlsbad, CA, USA; Cat. No. 10099-141) at 37 °C in a humidified incubator with 5% CO_2_/95% air. A γ-secretase enzyme inhibitor, *N*-[*N*-(3,5-difluorophenacetyl)-l-alanyl]-*S*-phenylglycine *t*-butyl ester (DAPT, Sigma, St. Louis, MO, USA; Cat. No. D5942), was utilized to suppress the activation of Notch signaling and the cells were divided into control group, oxygen glucose deprivation group (OGD group), oxygen glucose deprivation + DAPT (DAPT group) and oxygen glucose deprivation + hypertonic saline (HS group). At 1 h before hypoxia, the cells in DAPT group and HS group were treated with DAPT (10 µM) or HS (80 mM), respectively, in glucose free medium. The cells in the OGD group were incubated alone in the glucose free medium. After this, the treated cells from different groups were incubated in an air-tight hypoxia chamber with 3% O_2_/5% CO_2_ at 37 °C for 4 h. After OGD, to imitate the cerebral ischemia/reperfusion model in vivo, the cells in OGD, DAPT and HS group were incubated in a normoxia incubator for 1 h for re-oxygenation. The cells in the control group were maintained in DMEM-F12 supplemented with 10% FBS in an incubator under 5% CO_2_. Finally, the protein from the respective group was extracted from the BV-2 cells and stored at −80 °C for western blot analysis.

### Cell viability assay of BV-2 cells

To detect the effect of DAPT and HS on the viability of BV-2 cells, a Cell Counting Kit-8 (CCK-8, Dojindo China Co., Ltd., Shanghai, China, Cat. No. CK04) was used. The cells were plated into 96-well microplates (104 cells/well) and stabilized for 24 h. Then cells were divided into OGD + 10 µM DAPT (DAPT), OGD + different concentrations of HS (40, 60, 80, 100, 120, 140 and 160 mM). After this, the cells were subjected to OGD and re-oxygenation as described above. After that, 10 μL CCK-8 reagent was added to each well and incubated in a normoxia incubator at 37 °C for 4 h. The optical density was then read at 450 nm using a microplate reader. According to this test, the cell viability of BV-2 cells was not significantly changed with the HS concentration ranging from 40 to 80 mM (*P* > 0.05), but significantly decreased when the concentration of HS was over 100 mM (*P < 0.05), especially when the concentration of HS was over 120 mM (**P < 0.01). 10 μM DAPT had no significant effect on BV-2 cells viability (*P* > 0.05), even though it was reported to be able to inhibit Notch signaling pathway at this concentration [17]. The data are shown in Additional file 1. Therefore, 80 mM HS and 10 μM DAPT were chosen in this study and for all subsequent analysis.

### Western blotting

A total of 40 rats (n = 5 for each group) were used for western blotting. Total proteins from the peri-infarction cerebral cortex and BV-2 cells were extracted using a Total Protein Extraction Kit (Bei Jing Pu Li Lai Gene Technology Co., Ltd., Beijing China; Cat. No. P1053) following the manufacturer’s protocols. Protein concentrations were determined by a BCA Protein Assay kit (Shanghai Biocolor Bioscience and Technology Co. Ltd., Shanghai, China; PC0020). With addition of samples loading-buffer, protein samples were heated to 95 °C for 5 min and then equal amounts of protein were loaded on sodium dodecyl sulfate-polyacrylamide gel for electrophoresis. After electrophoresis, the protein was electroblotted onto polyvinylindene difluoride membrane and blocked with 5% non-fat dry milk in 0.1% tween-20 Tris-buffered saline for 1 h at room temperature. The membranes were then incubated with the following primary antibodies: Notch-1 (1:500, Cell Signaling Technology, Danvers, MA, USA; Cat. No. 4380), NICD (1:300, Cell Signaling Technology, Danvers, MA, USA; Cat. No. 4147), RBP-JK (1: 500, Abcam, Cambridge, MA, USA; Cat. No. ab180588), Hes-1 (1:500, Cell Signaling Technology, Danvers, MA, USA; Cat. No. 11988), iNOS (Santa Cruz Biotechnology, CA, USA; Cat. No. sc-650), Phos-NF-κB (1:1000, Cell Signaling Technology, Danvers, MA, USA; Cat. No. 3033) and β-actin (1:2000, Cell Signaling Technology, Danvers, MA, USA; Cat. No. 3700) overnight at 4 °C. After washing with 0.1% tween-20 Tris-buffered saline, the membranes were incubated with the goat anti-rabbit IgG-HRP (1:1000, Cell Signaling Technology, Danvers, MA, USA; Cat. No. 7074) or goat anti-mouse IgG-HRP (1:2000, Cell Signaling Technology, Danvers, MA, USA; Cat. No. 7076) secondary antibodies for 2 h at 4 °C. The immunoblots were developed by a chemiluminescence kit (BeiJing Pu Li Lai Gene Technology Co., Ltd., Beijing, China; P1010) and detected by an imaging densitometer (ImageQuant LAS 500, GE Healthcare Bio-Sciences AB, Uppsala, Sweden). Antibodies used in experiments is shown in Table 1. The relative density was quantified by FluorChem 8900 software (version 4.0.1, Alpha Innotech Corporation, San Leandro, CA, USA). In addition, the results were normalized by parallel western blots of β-actin and all the experiments were carried out in triplicate.

**Table 1:** Antibodies used for western blotting and staining

Antibody	Host	Source	Catalog number
Notch-1	Rabbit	CST, Danvers, MA, USA	4380
NICD	Rabbit	CST, Danvers, MA, USA	4147
RBP-JK	Rabbit	abcam, Cambridge, MA, USA	ab180588
Hes-1	Rabbit	CST, Danvers, MA, USA	11,988
iNOS	Rabbit	Santa Cruz Biotechnology, CA, USA	sc-650
Phos-NF-κB	Rabbit	CST, Danvers, MA, USA	3033
β-actin	Mouse	CST, Danvers, MA, USA	3700

### Double immunofluorescence labeling

At 24 h after reperfusion, 20 rats (n = 5 each group) were deeply anesthetized with 10% chloral hydrate and transcardially perfused with normal saline rapidly followed by 4% paraformaldehyde (Bei Jing Leagene Bioscience and Technology Co. Ltd., Beijing, China; DF0135). The brain was removed and the frozen coronal sections of 10 μm thickness were cut. The sections were washed 3 times with phosphate-buffered saline (PBS), and blocked with 5% bovine serum albumin (BSA) for 1 h at room temperature. After rinsing with PBS, the brain sections were incubated with the following primary antibodies: Notch-1 (1:100, Cell Signaling Technology, Danvers, MA, USA; Cat. No. 4380), NICD (1:100, Cell Signaling Technology, Danvers, MA, USA; Cat. No. 4147), RBP-JK (1: 100, Abcam, Cambridge, MA, USA; Cat. No. ab180588), Hes-1 (1:100, Cell Signaling Technology, Danvers, MA, USA; Cat. No. 11988), iNOS (1:100, Santa Cruz Biotechnology, Santa Cruz, CA, USA; Cat. No. sc-650), NF-κB (1:100, Cell Signaling Technology, Danvers, MA, USA; Cat. No. 3033) at 4 °C for overnight. On the following day, the sections were washed in PBS, and then incubated with the secondary antibodies Alexa Fluor^®^ 555 Goat Anti-Rabbit IgG (H + L) (1:200; Invitrogen Life Technologies Corporation; Carlsbad, CA, USA; Cat. No. A-21428) and FITC-conjugated Lectin (1:100, Sigma, St. Louis, MO, USA; Cat. No. L0401), which bind to microglia and blood vessel endothelial cells, for 2 h at room temperature. The nucleus was stained by 4,6-diamidino-2-phenylindole (DAPI) for 20 min. The sections were mounted with a fluorescent mounting medium and detected with a fluorescence microscope (Olympus DP73 Microscope, Olympus, Tokyo, Japan).

BV-2 cells seeded on the cover slips were fixed in 4% paraformaldehyde for 30 min, and then blocked by 5% BSA for another 30 min. Subsequently, the cover slips with the adherent cells were incubated with the primary antibodies as mentioned above at 4 °C overnight. After rinsing in PBS for 3 times the next day, the coverslips were incubated with the secondary antibodies and lectin as described above. Then the cover slips were incubated with DAPI for 10 min and mounted with a fluorescent mounting medium. All cover slips were viewed for labeled cells using a fluorescence microscope (Olympus DP73 Microscope, Olympus, Tokyo, Japan).

### Measurement of reactive oxygen species

The production of ROS in BV-2 cells of different groups was evaluated by the 5-(and-6)-chloromethyl-2,7-dichlorodihydrofluorescein diacetate probe (CM-H2DCFDA, Nanjing KeyGEN Biotech. CO., Ltd. Cat. No. KAGF018) following the manufacturer’s instruction, fluorescent images were taken by the fluorescent microscope (Nikon TI-S microscpe, Nikon, Tokyo, Japan).

### Data analysis

All data was analyzed by SPSS 13.0 using one-way analysis of variance (ANOVA). The intergroup comparisons were analyzed by the least-significant-difference (LSD) and the Student’s Newman–Keuls (SNK) tests. Differences were considered statistically significant if *P* < 0.05.

## Results

### 10% HS suppressed the protein expression of Notch signaling pathway in vivo

**Fig. 1:**
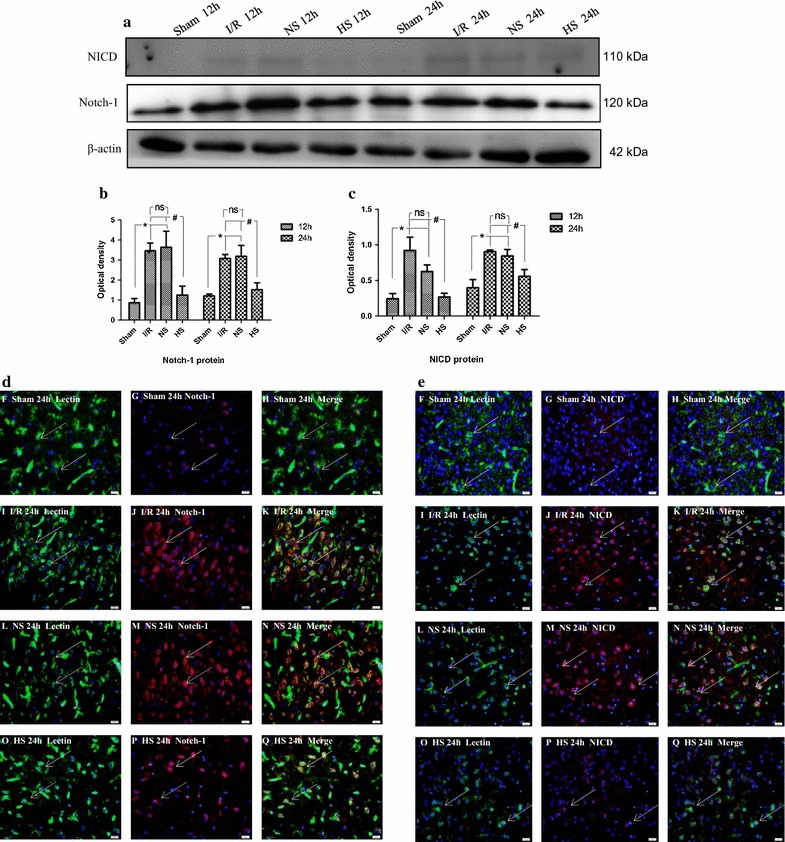
10% HS attenuates the expression of Notch-1 and NICD in peri-ischemic brain tissue. **a** The immunoreactive bands of Notch-1 (120 kDa), NICD (110 kDa), and β-actin (42 kDa), respectively. *Bar graphs* **b**, **c** show the protein expression of Notch-1 and NICD are significantly up-regulated in I/R and NS peri-ischemic brain tissues compared to the sham group (*P < 0.05) at 12 and 24 h; however, it is markedly attenuated following treatment with 10% HS at 12 h or 24 h (*#P* < 0.05). The values represent the mean ± SD in triplicate. **d** The immunofluorescence of Notch-1 (*G*, *J*, *M*, *P*, *red*), lectin^+^ microglia (*F*, *I*, *L*, *O*, *green*) and the co-localization of Notch-1 and microglia (*H*, *K*, *N*, *Q*). **e** The immunofluorescence of NICD (*G*, *J*, *M*, *P*, *red*), lectin^+^ microglia (*F*, *I*, *L*, *O*, *green*) and the co-localization of NICD and microglia (*H*, *K*, *N*, *Q*). The results also show that Notch-1 and NICD in activated microglia cells around the peri-ischemia cortex are markedly increased in I/R and NS rats and decreased following treatment with 10% HS for 24 h. *HS* hypertonic saline, *I/R* ischemic–reperfusion injury, *NS* normal saline, *NICD* Notch intracellular domain, *ns* no significant difference. *Scale bars* in *F*–*Q* 20 μm

**Fig. 2:**
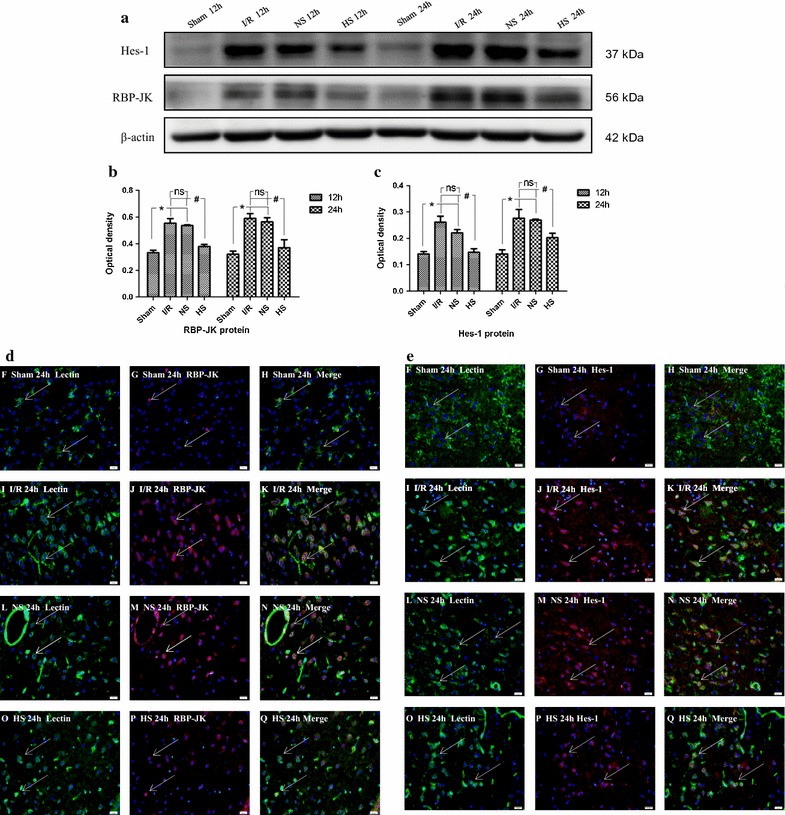
10% HS attenuates the expression of RBP-JK and Hes-1 in peri-ischemic brain tissue. **a** The immunoreactive bands of RBP-JK (56 kDa), Hes-1 (37 kDa) and β-actin (42 kDa), respectively. *Bar graphs* **b**, **c** show the protein expression of RBP-JK and Hes-1 are significantly up-regulated in I/R and NS peri-ischemic brain tissues compared to the sham group (*P < 0.05) at 12 and 24 h; however, it is markedly attenuated following treatment with 10% HS at 12 and 24 h (*#P* < 0.05). The values represent the mean ± SD in triplicate. **d** The immunofluorescence of RBP-JK (*G*, *J*, *M*, *P*, *red*), lectin^+^ microglia (*F*, *I*, *L*, *O*, *green*) and the co-localization of RBP-JK and microglia (*H*, *K*, *N*, *Q*). **e** The immunofluorescence of Hes-1 (*G*, *J*, *M*, *P*, *red*), lectin^+^ microglia (*F*, *I*, *L*, *O*, *green*) and the co-localization of Hes-1 and microglia (*H*, *K*, *N*, *Q*). Note that the expression of RBP-JK and Hes-1 in activated microglia cells around the peri-ischemia cortex are markedly increased in I/R and NS rats and decreased following treatment with 10% HS 24 h. *HS* hypertonic saline, *I/R* ischemic–reperfusion injury, *NS* normal saline, *RBP*-*JK* recombination signal sequence-binding protein J, *Hes*-*1* Hairy-Enhancer of Split-1. *ns* no significant difference. *Scale bars* in *F*–*Q* 20 μm

To explore the effects of 10% HS on the Notch pathway, the protein expression of members of Notch signaling pathway including Notch-1, NICD, RBP-JK and Hes-1 in the peri-ischemic cortex of ischemia rats given treatment of NS or 10% HS for 12 or 24 h was detected by western blotting. The results showed that the protein expression levels of Notch-1, NICD, RBP-JK and Hes-1 were significantly increased following ischemia–reperfusion (I/R) (*P < 0.05). The protein expression of Notch-1, NICD, RBP-JK and Hes-1 in the normal saline treated rats had no significant difference compared with the I/R rats (*P* > 0.05). On the contrary, the increased protein expression of Notch-1, NICD, RBP-JK and Hes-1 following I/R was significantly reduced with treatment of 10% HS at 12 and 24 h (#*P* < 0.05) (Figs. 1a–c, 2a–c).

### 10% HS suppressed the Notch signaling pathway in activated microglia following ischemia–reperfusion injury

To investigate whether 10% HS would suppress the Notch signaling pathway in microglia, expression of Notch-1, NICD, RBP-JK, Hes-1 in the microglia around the peri-ischemia cortex of I/R injury rats given NS or 10% HS treatment at 24 h was examined by double immunofluorescence. The immunofluorescence of Notch-1, NICD, RBP-JK, Hes-1 in activated microglia around the peri-ischemia cortex in I/R rats and NS rats was noticeably enhanced. Additionally, the immunofluorescence of Notch-1, NICD, RBP-JK, and Hes-1 was evidently reduced following treatment of 10% HS for 24 h (Figs. 1d, e, 2d, e).

### 10% HS inhibited iNOS expression in peri-ischemic cortex

**Fig. 3:**
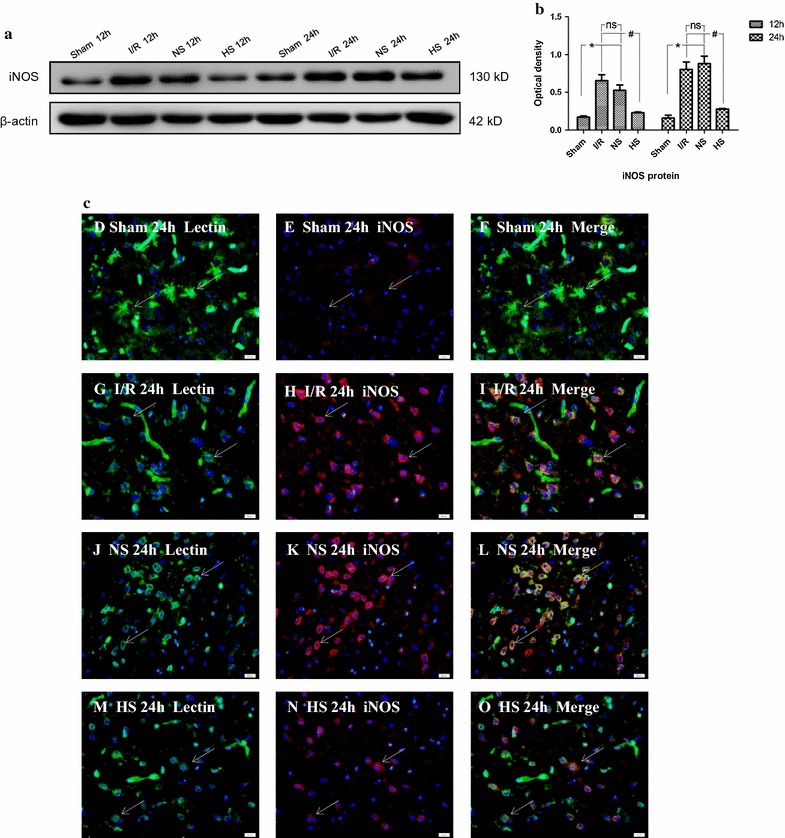
10% HS attenuates the expression of inflammatory mediator iNOS in peri-ischemic brain tissue. **a** iNOS (130 kDa) and β-actin (42 kDa) immunoreactive bands, respectively. **b** The protein expression of iNOS in I/R and NS rats is significantly up-regulated when compared to the sham group at 12 and 24 h after reperfusion (*P < 0.05); however, iNOS protein expression is drastically attenuated following treatment with 10% HS at 12 and 24 h when compares to I/R group and NS group (*P < 0.05); The values represent the mean ± SD in triplicate. Immunofluorescence images show the expression of iNOS (*red*) and lectin^+^ microglia (*green*) in the sham group (**D**–**F**) and in the penumbral zones of I/R group (*G*–*I*), NS group (*J*–*L*), and HS group (*M*–*O*) after reperfusion for 24 h. Note that there is a noticeable increase of iNOS expression in activated microglia around the peri-ischemia cortex in I/R and NS rats. however, iNOS expression is depressed in activated microglia following treatment with 10% HS 24 h. *HS* hypertonic saline, *I/R* ischemic–reperfusion injury, *NS* normal saline, *ns* no significant difference. *Scale bars* in *D*–*O* 20 μm

The protein expression of iNOS was significantly increased following I/R injury at 12 and 24 h compared to sham group (*P < 0.05), while no significant decrease was observed for the normal saline treatment group at either time point (*P* > 0.05). On the other hand, a significant reduction occurred with 10% HS treatment for 12 and 24 h compared to I/R and NS groups (#*P* < 0.05) (Fig. 3a, b). Immunofluorescence images showed a markedly increased expression of iNOS in activated microglia in peri-ischemia cortex of rats following I/R, and in rats given normal saline treatment. Very strikingly, iNOS expression in activated microglia was reduced with 10% HS treatment at 24 h (Fig. 3c).

### HS suppressed the Notch signaling pathway in hypoxia-activated BV-2 microglia in vitro

**Fig. 4:**
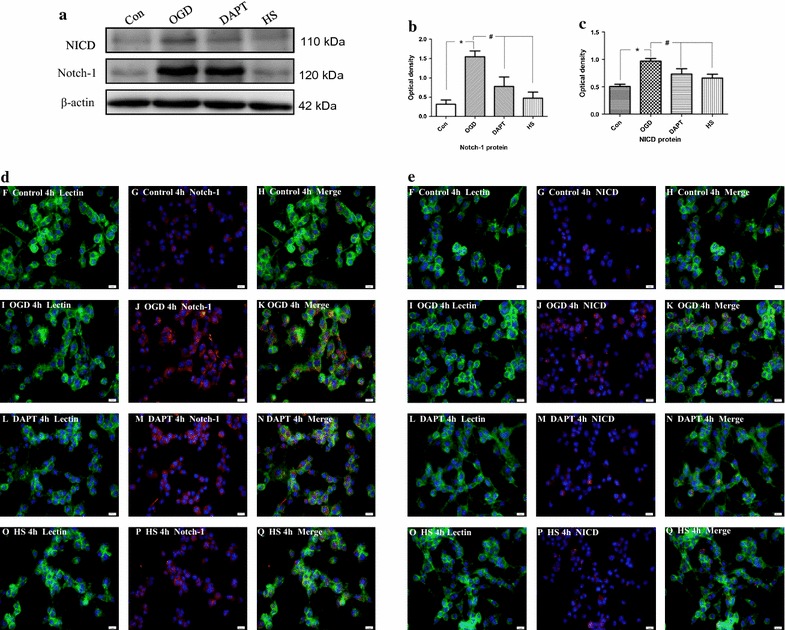
HS attenuates the expression of Notch-1 and NICD in hypoxia-activated BV-2 microglia. **a** The immunoreactive bands of Notch-1 (120 kDa), NICD (80 kDa) and β-actin (42 kDa), respectively. *Bar graph* **b** shows the protein expression of Notch-1 are significantly up-regulated in OGD BV-2 cells compared to control group (*P < 0.05); but it is moderately suppressed following treatment with 10 μM DAPT (*#P* < 0.05) and markedly suppressed following treatment with 80 mM HS (*#P* < 0.05). *Bar graph* **c** show the protein expression of NICD is significantly increased in OGD BV-2 cells compared to control group (*P < 0.05); however, it is decreased following treatment with 80 mM HS or 10 μM DAPT (*#P* < 0.05). The values represent the mean ± SD in triplicate. **d** The immunofluorescence of Notch-1 (*G*, *J*, *M*, *P*, *red*), lectin^+^ microglia (*F*, *I*, *L*, *O*, *green*) and the co-localization of Notch-1 in BV-2 microglia (*H*, *K*, *N*, *Q*). **e** The immunofluorescence of NICD (*G*, *J*, *M*, *P*, *red*), lectin^+^ microglia (*F*, *I*, *L*, *O*, *green*) and the co-localization of NICD in BV-2 microglia (*H*, *K*, *N*, *Q*). The results are consistent with the western blotting. Note that the expression of Notch-1 and NICD are increased in OGD-activated BV-2 cells and decreased with the treatment of 80 mM HS or 10 μm DAPT. *HS* hypertonic saline, *DAPT N*-[*N*-(3,5-difluorophenacetyl)-1-alanyl]-*S*-phenylglycine *t*-butyl ester, *OGD* oxygen glucose deprivation. *NICD* Notch intracellular domain. *Scale bars* in *F*–*Q* 20 μm

**Fig. 5:**
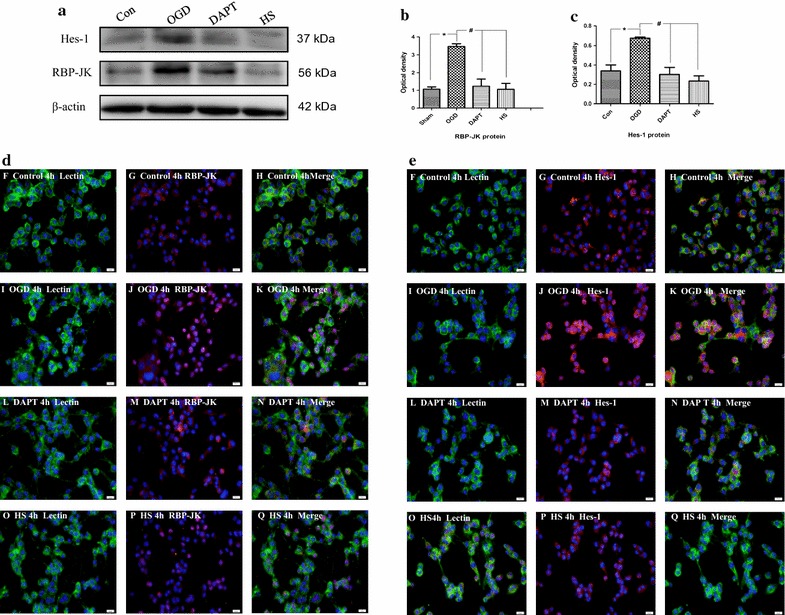
HS attenuates the expression of RBP-JK and Hes-1 in hypoxia-activated BV-2 microglia. **a** The immunoreactive bands of RBP-JK (56 kDa), Hes-1 (37 kDa) and β-actin (42 kDa), respectively. *Bar graph* **b**, **c** show the protein expression of RBP-JK and Hes-1 are significantly increased in OGD BV-2 cells compared to control group (*P < 0.05); but, it is decreased following treatment with 80 mM HS or 10 μM DAPT (*#P* < 0.05). The values represent the mean ± SD in triplicate. **d** The immunofluorescence of RBP-JK (*G*, *J*, *M*, *P*, *red*), lectin^+^ microglia (*F*, *I*, *L*, *O*, *green*) and the co-localization of RBP-JK in BV-2 microglia (*H*, *K*, *N*, *Q*). **e** The immunofluorescence of Hes-1 (*G*, *J*, *M*, *P*, *red*), lectin^+^ microglia (*F*, *I*, *L*, *O*, *green*) and the co-localization of Hes-1 in BV-2 microglia (*H*, *K*, *N*, *Q*). The results are aslo consistent with the western blotting. Note that the expression of RBP-JK and Hes-1 are increased in OGD-activated BV-2 cells and decreased with the treatment of 80 mM HS or 10 μm DAPT. *HS* hypertonic saline, *DAPT N*-[*N*-(3,5-difluorophenacetyl)-1-alanyl]-*S*-phenylglycine *t*-butyl ester, *OGD* oxygen glucose deprivation. *NICD* Notch intracellular domain, *RBP*-*JK* recombination signal sequence-binding protein J, *Hes*-*1* Hairy-Enhancer of Split-1. *Scale bars* in *F*–*Q* 20 μm

To investigate the effects of HS on activated microglia *in vitro*, BV-2 microglia cells were subjected to OGD for 4 h following the treatment of HS or DAPT. The protein expression of members of Notch signaling pathway including Notch-1, NICD, RBP-JK, and Hes-1 were significantly increased in BV-2 cells following OGD treatment (*P < 0.05). The protein expression level of Notch-1 was moderately depressed with the treatment of 10 μM DAPT and was markedly depressed with the treatment of 80 mM HS in OGD BV-2 cells (#*P* < 0.05). The protein expression of NICD, RBP-JK, and Hes-1 were significantly suppressed with the treatment of 80 mM HS or 10 μM DAPT in OGD-activated BV-2 cells (#*P* < 0.05) (Figs. 4a–c, 5a–c).

The immunofluorescence of Notch-1, NICD, RBP-jk, and Hes-1 was noticeably enhanced in OGD BV-2 microglia cells at 4 h; but the immunofluorescence of Notch-1, NICD, RBP-JK, and Hes-1 was suppressed in OGD BV-2 cells with the treatment of 80 mM HS or 10 μM DAPT (Figs. 4d, e, 5d, e).

### HS and DAPT reduced the expression of inflammatory cytokines Phos-NF-κB, iNOS and ROS in hypoxia-activated BV-2 microglia

**Fig. 6:**
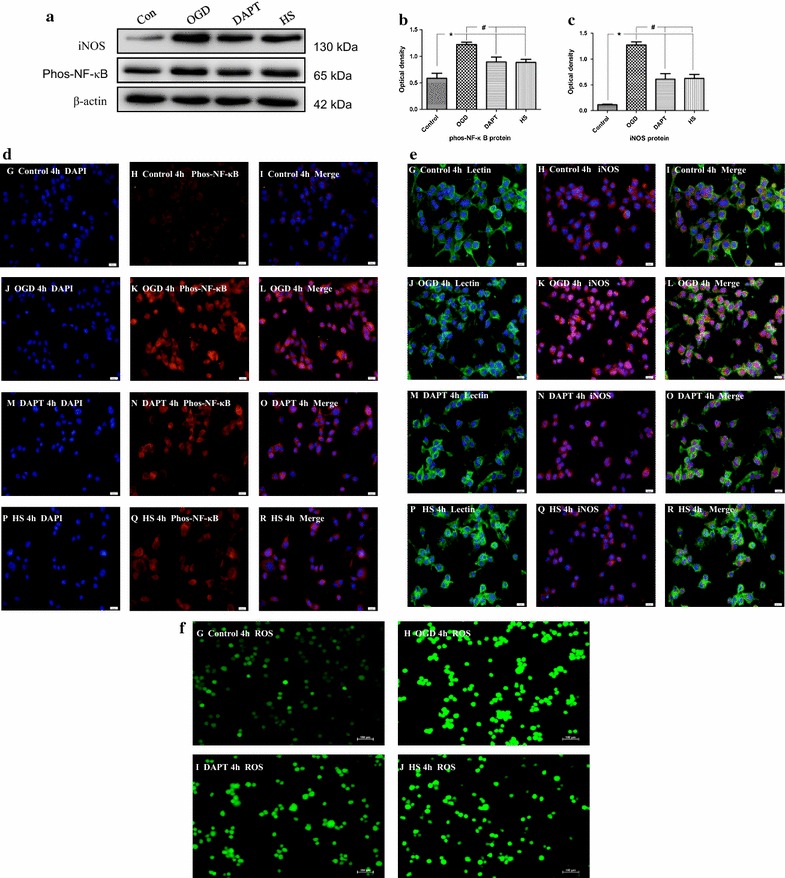
HS and DAPT reduced the expression of inflammatory mediators Phos-NF-κB, iNOS and ROS in hypoxia-activated BV-2 microglia. **a** The immunoreactive bands of Phos-NF-κB (65 kDa), iNOS (130 kDa), and β-actin (42 kDa), respectively. *Bar graph* **b**, **c** show the expression level of Phos-NF-κB and iNOS are significantly increased in OGD BV-2 cells (*P < 0.05) and significantly depressed following treatment with 80 mM HS or 10 μM DAPT (*#P* < 0.05). The values represent the mean ± SD in triplicate. **d** The immunofluorescence of Phos-NF-κB (*H*, *K*, *N*, *Q*, *red*) and its co-localization within the nucleus of BV-2 cells (*I*, *L*, *O*, *R*); **e** The immunofluorescence of iNOS (*H*, *K*, *N*, *Q*, *red*); lectin^+^ microglia (*G*, *J*, *M*, *P*, *green*) and the co-localization of lectin and iNOS (*I*, *L*, *O*, *R*); **f** shows the immunofluorescence of ROS (*G*, *H*, *I*, *J*, *green*); Note that the expression of Phos-NF-κB is markedly increased and translocated to the nucleus in OGD-activated BV-2 cells and decreased with the treatment of 80 mM HS or 10 μm DAPT. The expression of iNOS and ROS is also markedly increased in parallel with Phos-NF-κB in OGD BV-2 cells and depressed significantly following treatment with 80 mM HS or 10 μm DAPT. DAPI-blue; *HS* hypertonic saline, *DAPT N*-[*N*-(3,5-difluorophenacetyl)-1-alanyl]-*S*-phenylglycine *t*-butyl ester, *OGD* oxygen glucose deprivation. *Scale bars* in *F*–*Q* 20 μm

The changes of inflammatory mediators Phos-NF-κB, iNOS and ROS were evaluated in BV-2 microglia cells subjected to OGD for 4 h and following the treatment of 80 mM or 10 μM DAPT. The protein expression of iNOS and Phos-NF-κB was significantly increased (*P < 0.05); however, the expression level of iNOS and Phos-NF-κB was significantly depressed with the treatment of 80 mM or 10 μM DAPT (#*P* < 0.05) (Fig. 6a–c).

Phos-NF-κB expression was markedly enhanced and translocated to the nucleus in OGD BV-2 microglia cells at 4 h. It was evidently depressed in OGD BV-2 cells with the treatment of 80 mM HS or 10 μM DAPT. The immunofluorescence of iNOS and ROS was increased simultaneously with Phos-NF-κB in OGD BV-2 microglial cells at 4 h. Moreover, the expression of iNOS and ROS was suppressed with the treatment of 80 mM HS or 10 μM DAPT (Fig. 6d–f).

## Discussion

It is unequivocal from the present results that HS can modulate Notch signaling and thence suppress NF-κB-mediated proinflammatory cytokine production in activated microglia. Indeed, HS can suppress Notch signaling as manifested by the decreased protein expression of Notch-1, NICD, RBP-JK and Hes-1 in peri-ischemia area and OGD activated BV-2 cells. Our previous study suggested that HS can suppress the release of inflammatory mediators TNF-α and IL-1β in activated microglia and reduce the infarct size and brain water content following cerebral ischemia [26]. Here, we further extended the study showing that HS inhibits inflammatory mediator iNOS following cerebral ischemic–reperfusion injury at 12 and 24 h. Additionally, the expression of Phos-NF-κB, iNOS and ROS in OGD-activated BV-2 microglia was decreased with the treatment of 80 mM HS *in vitro*.

Recent studies reported that Notch signaling can be activated following brain injury [28] and is involved in the release of neuroinflammatory mediators in activated microglia [14]. Here, we show that Notch signaling pathway is activated as evidenced by the up-regulation of Notch-1, NICD, RBP-JK and Hes-1 in activated microglia in experimentally induced cerebral ischemia and OGD induced activated BV-2 microglia. An earlier report suggested that Notch-1 antisense mice exhibit significantly lower numbers of activated microglia and reduced proinflammatory cytokine expression in the ipsilateral ischemic cortices compared to non-Tg mice [29]. Other studies showed that inhibition of Notch pathway reduced the damage to brain cells and improved functions. Notch can endanger neurons by modulating pathways that increase their vulnerability to apoptosis, and by activating microglial cells and stimulating the infiltration of proinflammatory leukocytes [15, 16, 30]. In the present study, we show that when Notch signaling is inhibited by DAPT (a γ-secretase inhibitor), the expression of Phos-NF-κB, iNOS and ROS was significantly decreased. It is evident that DAPT can block the Notch signaling as shown by significantly reduction in NICD protein levels and expression of RBP-JK, Hes-1 expression in BV-2 microglia.

Furthermore, we have shown that HS suppresses the activation of Notch signaling and attenuates expression of members of Notch signaling in activated microglia in peri-ischemia tissue and hypoxic BV-2 Microglia. Additionally, we have shown by double immunofluorescence results that the nuclear translocation of NICD, RBP-JK and Hes-1 is down-regulated in activated microglia following treatment of HS *in vivo* and *in vitro*. This suggests that HS disrupts Notch signaling not only by down-regulating the Notch receptor but also the activation of Notch signaling. During the maturation of Notch1 protein, a 300-kDa precursor molecule of Notch receptor is constitutively cleaved by furin-like convertase [31]. Once Notch receptor binds to its ligand, the Notch signaling will be activated by two protease hydrolysis process: i) the first one is catalyzed by an ADAM-type metalloprotease at the extracellular part of Notch receptor; ii) the second one is catalyzed by γ-secretase containing a complex at the transmembrane region that releases the NICD which represents the activation of Notch signaling [11, 12]. It is suggested that HS may act on furin-like convertase thus suppressing the expression of Notch-1 and may act through the two key enzymes (ADAM-type metalloprotease, γ-secretase) thus inhibiting the expression of NICD, RBP-JK and Hes-1.

It has been suggested that Notch and NF-κB pathways operate synergistically in microglia activation and function [32, 33]. It is well known that the function of microglia is defensing of neural parenchyma, removing cellular debris not only during normal development but also in pathological conditions; in addition to scavenging function, activated-microglia exert a robust inflammatory response [34–36]. NF-κB is widely thought to be an important regulator of neuroinflammatory and is crucial in cerebral ischemia [37–39]. Activated NF-κB is translocated to the nucleus to combine with proinflammatory genes such as TNF-α, IL-1β, IL-6, IL-8, iNOS and cyclo-oxygenase-2, which consequently results in expanded inflammatory reaction [40]. Transgenic mice deficient in the NF-κB subunit or pharmacological inhibition of the NF-κB pathway develop significantly smaller infarcts after middle cerebral artery occlusion [41, 42]. Some studies have considered that Notch is the upstream of NF-κB [16, 17]. It has been reported that γ-secretase-mediated Notch signaling acts to keep NF-κB activity and release of inflammatory in peripheral T cells; inhibition of Notch with pharmacological attenuates the nuclear distribution of NF-κB, thus reducing transcriptional activity [43]. On the other hand, Notch signaling can up-regulate the expression of the active NF-κB signaling components, thus enhancing NF-κB activation [44]. Here, we showed that the protein expression and nuclear translocation of NF-κB was significantly down-regulated in BV-2 microglia when Notch signaling was inhibited by DAPT.

In addition, we show that HS can inhibit the expression of NF-κB by Western blot; the nuclear translocation of NF-κB is also down-regulated with treatment of 80 mM HS in OGD-activated microglia by immunofluorescence. In parallel to NF-κB, the expression of iNOS and ROS was significantly decreased with the treatment of HS. Inflammatory mediators are thought to be vital in acute and chronic brain injury, such as stroke [45], Alzheimer’s disease [46] and Parkinson’s disease [47]. Excessive release of proinflammatory mediators can lead to neuronal death, glia activation, synaptic impairment and exacerbate neurodegenerative disorders. iNOS and ROS are known to be involved in oxidative stress that was indicated to contribute to the death of neurons and glia [48, 49]. Activated microglial cells are thought to be the main origin of inflammatory mediators and crucially involved in neuronal damage in the penumbra in ischemia stroke [5–7]. Our previous study had shown that HS could suppress the release of inflammatory mediators, namely, TNF-α and IL-1β in activated microglia [26]. Here, we show that HS can suppress the release of NF-κB mediated iNOS and ROS. It is relevant to note that hypertonic treatment decreases I-kBα phosphorylation and thus decreases NF-κB nuclear translocation and activation [25]. In light of present finding, it is suggested that HS inhibits production of NF-κB mediated proinflammatory mediators in activated microglia by disrupting the Notch signaling.

As far as can be ascertained, this is the first report which demonstrates that HS participates in Notch-dependent inflammatory changes associated with cerebral ischemia. Arising from the above, we suggest that HS can be helpful in diminishing the neuronal damage caused by excessive neuroinflammation following ischemia–reperfusion injury. Furthermore, because inflammation mediators are also implicated in other brain damages such as disruption of blood-brain barrier, it stands to reason that hypertonic saline would also be beneficial to restore these functions that are expected to be compromised in cerebral ischemic. Thus, HS could be a potential therapeutic option for amelioration of microglia–mediated neuroinflammation and thence improving neurological function in cerebral ischemia.

## Conclusion

We show here that HS can suppress Notch signaling and proinflammatory mediators such as Phos-NF-κB, iNOS and ROS in activated microglia. Pharmacological inhibition of Notch signaling attenuates the expression of Phos-NF-κB, iNOS and ROS. In consideration of the present results along with others, it is suggested that HS may suppress of inflammatory mediators following ischemia/hypoxic through the Notch signaling which operates synergistically with NF-κB pathway in activated microglia. Our study has added new evidence that HS can attenuate inflammation and this can be neuroprotective in cerebral ischemia, thus adding a morphological basis at the cellular level for clinicians in the use of hypertonic saline in patients with an ischemic stroke.

## Additional file

**Additional file 1.** The effect of HS and DAPT on BV-2 cell viability.ppt. Panel A shows 40 mM HS, 60 mM HS, and 80 mM HS have no significant effect on BV-2 cell viability compared to OGD group (*P* > 0.05); but the BV-2 cell viability was significantly decreased in 100 mM HS (**P* < 0.05), 120 mM HS, 140 mM HS, and 160 mM HS (***P* < 0.01) compared to OGD group. Panel B shows that 10 μM DAPT has no significant effect on BV-2 cell viability compared to OGD group (*P* > 0.05). The values represent the mean ± SD in triplicate. HS, hypertonic saline; DAPT, N-[N-(3,5-difluorophenacetyl)-1-alanyl]-S-phenylglycinet-butyl ester; OGD, oxygen glucose deprivation. ns, no significant difference.

## Abbreviations

BSA: bull serum albumin
CCK-8: Cell Counting Kit-8
CCA: common carotid artery
CNS: central nervous system
DAPT: *N*-[*N*-(3,5-difluorophenacetyl)-1-alanyl]-*S*-phenylglycine *t*-butyl ester
DMEM-F12: Dulbecco’s modified Eagle medium/nutrient mixture F-12
DAPI: 4,6-diamidino-2-phenylindole
ECA: external carotid artery
FBS: fetal bovine serum
HRP: horseradish peroxidase
HS: hypertonic saline
Hes-1: Hairy-Enhancer of Split-1
HERP: HES-related proteins
ICA: internal carotid artery
I/R: ischemic–reperfusion injury
ICH: intracranial hemorrhage
ICP: intracranial pressure
IL-1β: interleukin-1 beta
iNOS: inducible nitric oxide synthase
MCA: middle cerebral artery
MCAO: middle cerebral artery occlusion
NS: normal saline
NICD: Notch intracellular domain
NO: nitric oxide
OGD: oxygen glucose deprivation
PBS: phosphate buffered saline
RBP-JK: recombination signal sequence-binding protein J
ROS: reactive oxygen species
SD: Sprague-Dawley
TBI: traumatic brain injury
TNF-α: tumor necrosis factor alpha
