# Supplementary figures and images for: Hypertonic saline attenuates expression of Notch signaling and proinflammatory mediators in activated microglia in experimentally induced cerebral ischemia and hypoxic BV-2 microglia
Source: BMC Neurosci. 2017 Mar 14;18:32. doi: 10.1186/s12868-017-0351-6 (PMC5348816; doi:10.1186/s12868-017-0351-6)

## Slide 1
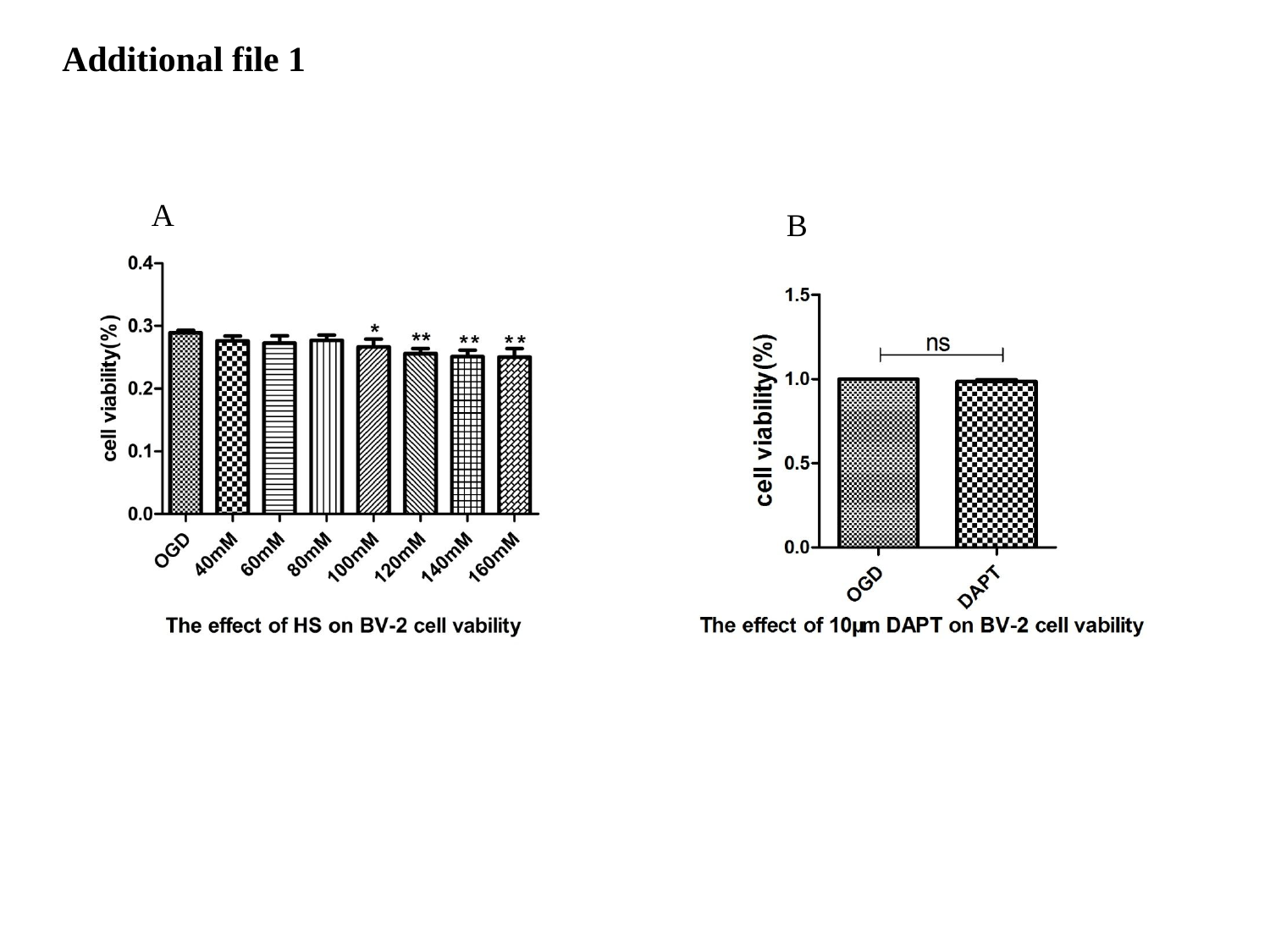

Additional file 1
A
B

Supplement: Supplementary file 1 — Additional file 1. The effect of HS and DAPT on BV-2 cell viability.ppt. Panel A shows 40 mM HS, 60 mM HS, and 80 mM HS have no significant effect on BV-2 cell viability compared to OGD group (P > 0.05); but the BV-2 cell viability was significantly decreased in 100 mM HS (*P < 0.05), 120 mM HS, 140 mM HS, and 160 mM HS (**P < 0.01) compared to OGD group. Panel B shows that 10 μM DAPT has no significant effect on BV-2 cell viability compared to OGD group (P > 0.05). The values represent the mean ± SD in triplicate. HS, hypertonic saline; DAPT, N-[N-(3,5-difluorophenacetyl)-1-alanyl]-S-phenylglycinet-butyl ester; OGD, oxygen glucose deprivation. ns, no significant difference. [file 12868_2017_351_MOESM1_ESM.ppt]
